# Achieving Aesthetics and Function in Class III Malocclusion Through Orthodontic Camouflage: A Clinical Case Report

**DOI:** 10.7759/cureus.65063

**Published:** 2024-07-21

**Authors:** Rizwan Gilani, Anjali Kathade, Shefali Singh, Aishwarya R Atey

**Affiliations:** 1 Department of Orthodontics and Dentofacial Orthopedics, Sharad Pawar Dental College, Datta Meghe Institute of Higher Education and Research, Wardha, IND

**Keywords:** intermaxillary elastics, dental alignment, facial aesthetics, non-surgical treatment, orthodontic camouflage, class iii malocclusion

## Abstract

Class III malocclusion is a challenging dental and skeletal condition characterized by a protrusive mandible, retrusive maxilla, or a combination of both. Treatment options include growth modification, orthodontic camouflage, and orthognathic surgery. While surgery often provides definitive results for severe cases, orthodontic camouflage is a viable alternative for managing mild to moderate skeletal discrepancies in adults. This case report illustrates the successful use of orthodontic camouflage in a 19-year-old female with skeletal and dental class III malocclusion, emphasizing nonsurgical strategies to achieve functional and aesthetic improvements. The patient presented with concerns about her bite and facial profile. Clinical examination revealed a concave profile, prominent mandible, and class III molar and canine relationships with a negative overjet. The radiographic analysis confirmed a skeletal class III relationship (ANB angle of -2°) and normal vertical growth patterns. The chosen nonsurgical treatment plan involved fixed orthodontic appliances and class III intermaxillary elastics to correct the malocclusion and improve facial aesthetics. The treatment phases included initial alignment, class III elastic application to adjust the occlusion, and detailed finishing to refine results. After 20 months, the treatment resulted in a positive overjet, class I molar and canine relationships, and improved facial aesthetics with reduced mandibular prominence. The patient expressed satisfaction with both functional and aesthetic outcomes. This case demonstrates that orthodontic camouflage can effectively manage mild to moderate class III malocclusion in non-growing patients. Successful outcomes depend on precise treatment planning, patient compliance, and regular monitoring. While surgical options remain necessary for severe cases, orthodontic camouflage provides a less invasive alternative for suitable patients, significantly improving dental function and facial aesthetics.

## Introduction

Class III malocclusion is quite a remarkable dental condition specifically characterized by the presence of a protruding mandible, retrusive maxilla, or a combination of these and associated all the components. Malocclusion being a complex one makes it more complicated to diagnose and find the right etiology for the present condition and to accordingly make a customized treatment plan for an individual [1]. A wide variety of treatment options are available for the treatment of class III malocclusion such as orthognathic surgery, orthodontic camouflage, or growth modulation may be used to help patients with class III malocclusion achieve improved facial aesthetics and appropriate occlusion. To plan a treatment for a patient a lot of things have to be taken into consideration, the age of the patient, the severity of the malocclusion present, what is the main concern for the patient, and all the clinical and radiographic analysis that will influence the patient’s outcome [2].

The management of the malocclusion can be done according to the age of the patient, as the growth modulation could be done in patients who have not yet reached puberty, and in those cases, growth modulation could be beneficial to the patient, while orthodontic surgery is typically reserved for patients who have reached adulthood and needs orthodontic camouflage. The decision between surgical and non-surgical alternatives for adult patients is guided by the degree of malocclusion and certain cephalometric parameters, such as Incisor Mandibular Plane Angles and ANB [3]. Factors such as the Wits assessment, SN, maxillary/mandibular ratio, and lower gonial angle can be incorporated into a predictive model to help distinguish between instances that can be treated with orthodontics alone and those that need surgery. Differentiating between situations that are borderline surgical and orthodontic is still difficult to make and making a decision of intervention by surgery and camouflage is quite a job.

Class III malocclusion is more predominant in Asian populations compared to Western countries, adding another layer of complexity in diagnosis and treatment. There are three main treatment strategies: growth modification, dentoalveolar compensation (orthodontic camouflage), and orthognathic surgery [4]. Each approach has its indications and limitations, with growth modification needing to start before the end of the pubertal growth spurt. For nongrowing patients, orthodontic camouflage improves dental occlusion and appearance, while combined orthodontic and orthognathic surgery addresses both functional and aesthetic concerns.

Evaluating treatment outcomes is crucial for evidence-based practice. Studies indicate that patients undergoing surgical mandibular advancement often report higher satisfaction compared to those receiving orthodontic camouflage [5]. The extent of the initial discrepancy plays a noteworthy role in the perceived success of the treatment, with larger discrepancies typically necessitating surgical intervention. Patients' expectations may not always be met by orthodontic camouflage, especially if they have unique facial features. In light of the implications for the overall appearance and face profile, simulation studies support the surgical approach [6].

In orthodontic practice, choosing between orthodontic camouflage surgery (orthodontia) and treating class III malocclusion is still an inevitable and judgmental difficulty. Intermaxillary elastics and other orthodontic materials are still developing, which helps to support these treatments and improve patient outcomes and their efficacy. To provide patients with class III malocclusion the best care possible, practitioners must comprehend and navigate these challenges [7].

## Case presentation

**Figure 1:**
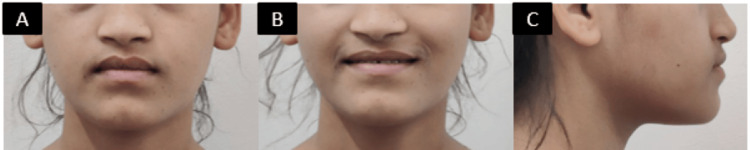
(A) Frontal, (B) smiling, and (C) profile pictures.

A 19-year-old female presented with concerns about her bite and facial appearance, specifically noting issues with her dental alignment and the prominent position of her lower jaw (Figure 1).

**Figure 2:**
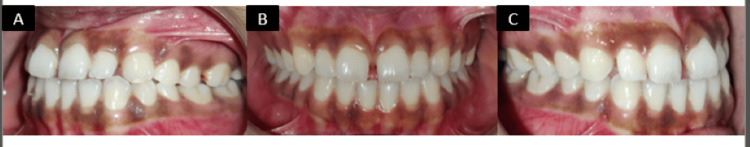
(A) Left buccal, (B) frontal, and (C) right buccal.

She had no significant medical history and attended regular dental checkups, maintaining good oral health. Upon clinical examination, the patient exhibited a concave facial profile with a prominently positioned mandible, characteristic of a skeletal class III relationship. Intraoral examination revealed a class III malocclusion, where the lower molars and canines were mesially positioned relative to the upper teeth. She had a negative overjet of 0 mm, indicating no horizontal overlap between the upper and lower front teeth, and an edge-to-edge bite without vertical overlap (Figure 2).

**Figure 3:**
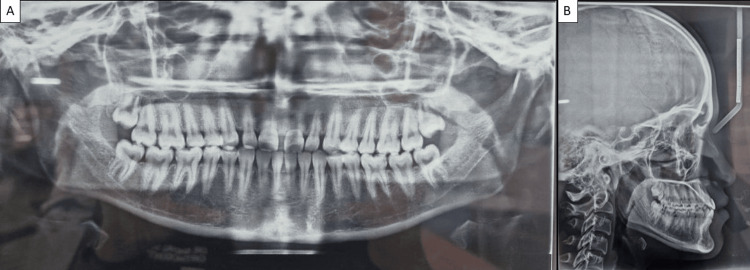
(A) Pretreatment OPG and (B) pretreatment lateral cephalogram. OPG, orthopantamogram

The radiographic analysis included a lateral cephalogram, which confirmed the skeletal class III relationship with an ANB angle of -2°, an SNB angle of 80°, and an SNA angle of 78° (Figure 3).

**Figure 4:**
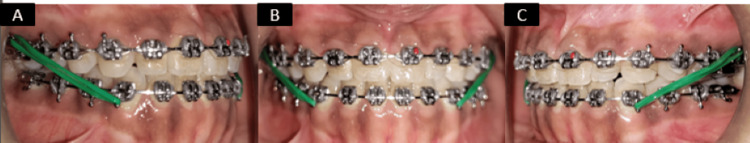
Inter-maxillary elastic shown: (A) right buccal, (B) frontal, and (C) left buccal.

The mandibular plane angle was normal, suggesting standard vertical growth patterns. The orthopantomogram (OPG) showed no pathology, and all permanent teeth were present, including the third molars. The diagnosis was a skeletal and dental class III malocclusion with a negative overjet. The treatment plan aimed to correct the class III malocclusion, achieve a positive overjet, and improve facial aesthetics without surgical intervention. The proposed approach involved the use of fixed orthodontic appliances (braces) on both the upper and lower arches, supplemented with class III intermaxillary elastics (3/16-inch, 6 oz) to encourage maxillary forward movement and mandibular backward positioning. The treatment was divided into several phases. The initial alignment and leveling phase (0-6 months) involved bonding brackets and using light continuous arch wires to level and align both arches. Progression from 0.016 round NiTi wires to 0.016 x 0.022 NiTi wires, and finally to 0.016 x 0.022 stainless steel (SS) wires, achieved noticeable improvement in arch alignment. From months 6 to 18, class III elastics were introduced to further correct the occlusion. During this period, the patient’s compliance with elastic wear was closely monitored, leading to the development of a positive overjet. The continued use of class III elastics refined the occlusion and corrected the midline (Figure 4).

**Figure 5:**
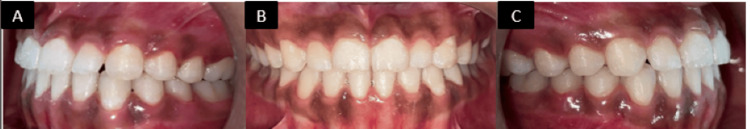
Posttreatment intraoral photographs: (A) right buccal with class I molar and canine relation; (B) frontal view with the midline coinciding and positive overjet and overbite; (C) left buccal with class I molar and canine relation.

The finishing and detailing phase (final two months) involved precise adjustments to perfect the occlusion. Braces were removed, and fixed retainers were placed in both the upper and lower arches from canine to canine to maintain the results. The outcome of the treatment was successful, a positive overjet of 2 mm was achieved, and the occlusal relationships were corrected to class I for both molars and canines (Figure 5).

**Figure 6:**
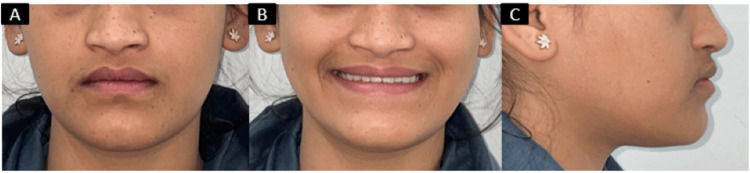
Posttreatment photographs: (A) frontal, (B) smiling, and (c) right profile.

The patient's facial profile improved with a reduction in mandibular prominence, enhancing her overall facial aesthetics. The patient reported satisfaction with both the functional and aesthetic results of the treatment (Figure 6).

**Figure 7:**
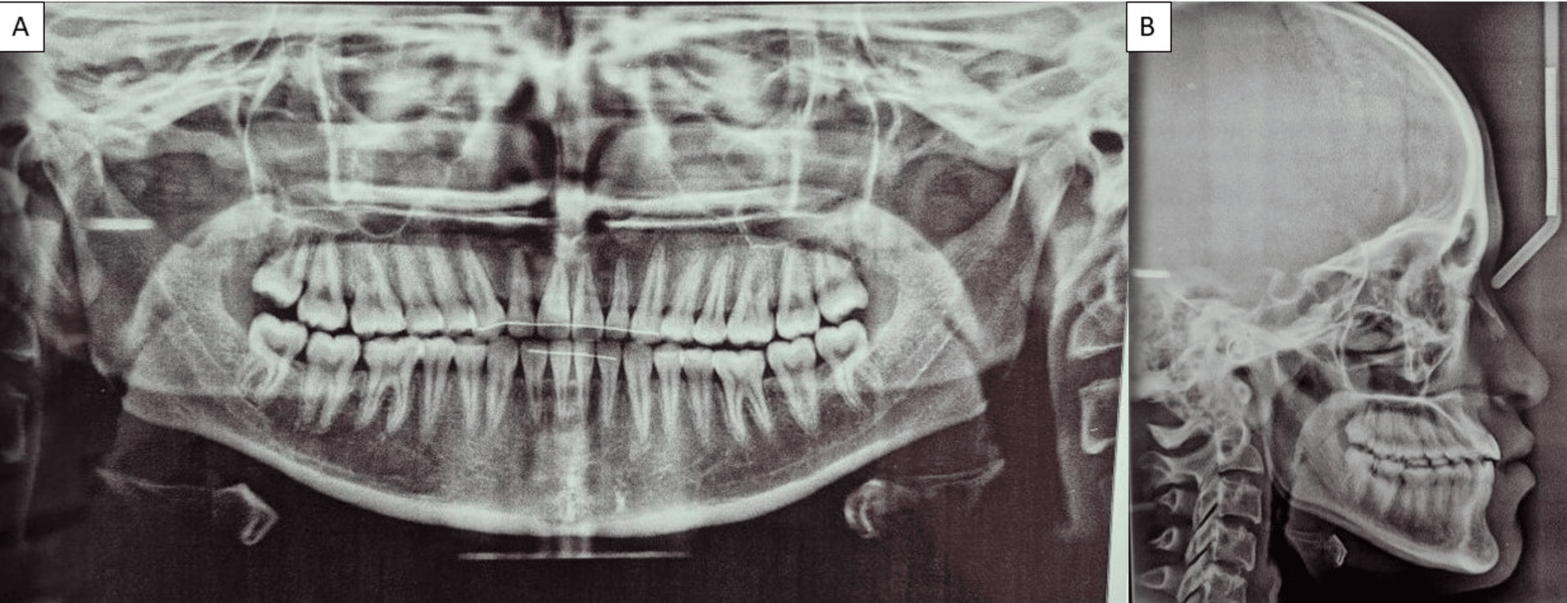
(A) Posttreatment OPG with fixed retainer given and (B) posttreatment lateral cephalogram with positive overjet. OPG, orthopantamogram

Posttreatment, the patient was scheduled for regular follow-ups at three-month intervals to monitor the retention and stability of the treatment results. She was also instructed on the importance of consistent retainer use and maintaining excellent oral hygiene practices (Figure 7).

This case demonstrates the effective use of nonsurgical orthodontic treatment to correct a class III malocclusion using intermaxillary elastics. The success of the treatment was significantly influenced by the patient’s compliance with elastic wear and the regular follow-ups that allowed for necessary adjustments to the treatment plan. While nonsurgical approaches may not be suitable for all class III cases, particularly those with severe skeletal discrepancies, this case illustrates the potential for achieving significant improvements in both dental function and facial aesthetics through dedicated orthodontic mechanics and patient cooperation.

## Discussion

Class III malocclusion, represented by an anterior relationship of the lower arch corresponding to the upper arch, presents a remarkable challenge in orthodontics, especially in adult patients where growth modification can’t be done. Orthognathic surgery is typically the preferred course of action to address underlying skeletal abnormalities while treating skeletal class III malocclusion in adults. Some individuals cannot afford surgery because of their financial problems, health, or any other circumstantial difficulties. This case presentation examines various orthodontic camouflage techniques and how well they work to treat *mild-to-moderate *skeletal class III malocclusions without the need for surgical intervention. Orthodontic camouflage includes the strategic movement of teeth to compensate for skeletal discrepancies present, thereby creating an acceptable functional and aesthetic outcome without any alterations made to the skeletal structure. This approach can be particularly beneficial for adults who have mild to moderate skeletal discrepancies and seek to avoid the complexities of surgical correction.

In a study by Bhandari et al., a case was presented in which an adult with class III skeletal malocclusion was treated using a unique approach in orthodontics. The team decided to treat the case by orthodontic camouflage with the decision to extract the first premolar and the lower incisors. The objective of this approach was to enhance the occlusion as well as facial aesthetics. This is one of the most logical orthodontic strategies and simplest for dealing with *mild-to-moderate* malocclusions in patients with skeletal discrepancies; it achieves firm results that remain pleasing even when surgery is not done [8].

Research done by Araujo and Squeff showed us how efficient the camouflage treatment for skeletal class III malocclusion is. They particularly emphasized the adults who had a slight to moderate number of dental disproportions and malalignment to correct, and it had an impact on both the function and appearance of the patient. Also, this method is beneficial in cases where a patient is unable to bear such a large amount needed to undergo surgical intervention for the corrections. This modality of change in the teeth position, and dentofacial deformities which eventually balances the occlusion, and bites makes a harmonious facial profile [9].

Seo et al. presented a case report on a young patient with class III malocclusion, where they used palatal mini-implants for rapid maxillary expansion (RME) and mandibular distalization. This approach by the researchers resulted in remarkable tooth movement, achieving a class I occlusion and improved facial symmetry. Bone-borne rapid maxillary expansion (BBRME) and class III elastics showed how new inventions correct the discrepancies in growing individuals without surgical interventions and lead to stable and visually satisfying outcomes [10].

A remarkable case was presented by Bellot-Arís et al. about a nonsurgical approach to the correction of class III malocclusion, where they combined the fixed appliance and the skeletal anchorage to correct issues like posterior crossbite and an anterior edge-to-edge bite. This plan of action included first expanding the arch width using a lingual archwire. Mini screws were then used for the retraction of the teeth. This plan of treatment shows that precise planning helps in executing the various modalities of treatment of complex cases where the surgical approach is found to be the management but can be done without the need for surgery as well [11].

## Conclusions

Orthodontic camouflage is a viable and effective approach to surgical intervention for management of mild-to-moderate skeletal class III malocclusion in adults. After careful case selection and a proper diagnosis with precise treatment planning and the application of advanced orthodontic techniques, dentists can achieve remarkable improvements in occlusion and facial aesthetics. While orthognathic surgery remain as the definitive solution for severe skeletal discrepancies, orthodontic camouflage offers a less invasive, patient-preferred option that can provide stable and satisfactory results in suitable cases.
